# Adrenal Infarction in Pregnancy Secondary to Elevated Plasma Factor VIII Activity

**DOI:** 10.7759/cureus.19491

**Published:** 2021-11-11

**Authors:** Reshmi Mathew, Aleem Ali, Kimberly Sanders, Andrew Flint, Sanjay Lamsal, Heather DeReus, Micaela Cueno, Rafik Jacob

**Affiliations:** 1 Internal Medicine, University of Florida College of Medicine, Jacksonville, USA; 2 General Internal Medicine, University of Florida College of Medicine, Jacksonville, USA; 3 Radiology, University of Florida College of Medicine, Jacksonville, USA

**Keywords:** pai, elevated factor viii, acute abdomen, pregnancy, unilateral adrenal infarction

## Abstract

Unilateral adrenal infarction is a rare cause of acute abdomen in pregnancy (AAP). Its presentation is non-specific and requires a high index of suspicion with a low threshold to obtain radiographic imaging for diagnosis. Evaluating AAP is challenging as diagnostic radiographic imaging is often limited in relation to radiation exposure to the developing fetus. We describe a case of a 24-year-old pregnant female who presented with severe acute abdominal pain. The patient’s pain was refractory to intravenous analgesics and ultrasonography was inconclusive. Computed tomography (CT) scan was not obtained due to the risk of radiation exposure to the developing fetus. Due to the persistence of pain and suspicions for other serious etiologies, magnetic resonance imaging (MRI) was completed and the patient was diagnosed with acute unilateral adrenal infarction. In this case report, unilateral adrenal infarction was likely secondary to elevated plasma factor VIII levels. Even with the physiological elevation of factor VIII levels during pregnancy, levels greater than 150 IU/dL confer greater than five-fold increased risk of venous thrombosis. Once hemorrhage is excluded, patients should be started on therapeutic anticoagulation to prevent progression of adrenal infarct or infarction of the contralateral adrenal gland. Prompt recognition and treatment of acute adrenal infarction during pregnancy are of paramount importance to prevent adverse outcomes for both the mother and fetus.

## Introduction

The diagnosis of acute abdomen in pregnancy (AAP) involves unique diagnostic and therapeutic challenges. The diagnosis can be challenging due to altered anatomy secondary to an enlarged uterus, physiological changes associated with pregnancy, and the limited use of radiological modalities due to fear of exposing the fetus to radiation [1]. The nonspecific presentations of AAP may lead to delays in diagnosis and treatment that put both the mother and fetus at risk of fatal outcomes.

The etiology of AAP is extensive and can be classified into obstetric and non-obstetric causes. The differential for AAP includes ectopic pregnancy, placental abruption, acute fatty liver of pregnancy, hemolysis, elevated liver enzymes, and low platelet count (HELLP) syndrome, ovarian torsion, or uterine rupture. Common non-obstetric causes of AAP include acute appendicitis, cholecystitis, pancreatitis, and pyelonephritis. Adrenal infarction is a rare non-obstetric cause of AAP and one should maintain a high index of suspicion for this diagnosis [2,3].

Acute adrenal infarction usually presents with sudden, severe, and unremitting flank pain. Moreover, some patients may present with hypotension and hyponatremia due to subsequent primary adrenal insufficiency (PAI). The most common cause of adrenal infarction is adrenal vein thrombosis, and its occurrence warrants hematological evaluation [4]. In this case report, we will discuss elevated plasma factor VIII activity as a rare cause of acute adrenal infarction in a 24-year-old pregnant female at 29 weeks of gestation.

## Case presentation

A 24-year-old pregnant female, gravida 4 para 1, at 29 weeks of gestation presented to the emergency room with acute severe unremitting left flank pain. Her medical history revealed four prior pregnancies with three full-term spontaneous vaginal deliveries and one first trimester miscarriage. The patient reported no previous use of hormonal contraception and was not taking any medications. Pertinent family history was significant for systemic lupus erythematosus in her mother with recurrent deep vein thrombosis and transient ischemic attacks. In addition, recent medical history revealed a hospitalization to an outside hospital two weeks prior, after presenting with similar left flank pain which was treated as urosepsis with empiric intravenous antibiotics. The patient was discharged on oral antibiotics with resolution of abdominal pain. On this admission, the patient endorsed recurrent left upper quadrant and flank pain described as “the worst pain of my life,” associated with nausea and vomiting. Her vital signs were as follows: pulse was 105 beats/min, temperature was 35.9°C (96.6°F), blood pressure (BP) was 115/60 mmHg, respiratory rate (RR) was 24 breaths/min, oxygen saturation (SpO_2_) was 97% on 2L nasal cannula (NC). Laboratory evaluation included a complete blood count with hemoglobin level at 11.9 g/dL and platelet count was normal (394 x 10^3^/L). White blood cell count was elevated at 16.35 cells/uL (81% neutrophils, 12.3% lymphocytes, 4.9% monocytes, 0.5% eosinophils, 0.2% basophils). Liver function tests, amylase, and lipase were all within normal limits. Urine analysis was unrevealing. Abdominal ultrasound was negative for acute pathology; however, the study was limited by the patient’s significant pain to sonographic probe pressure.

**Figure 1:**
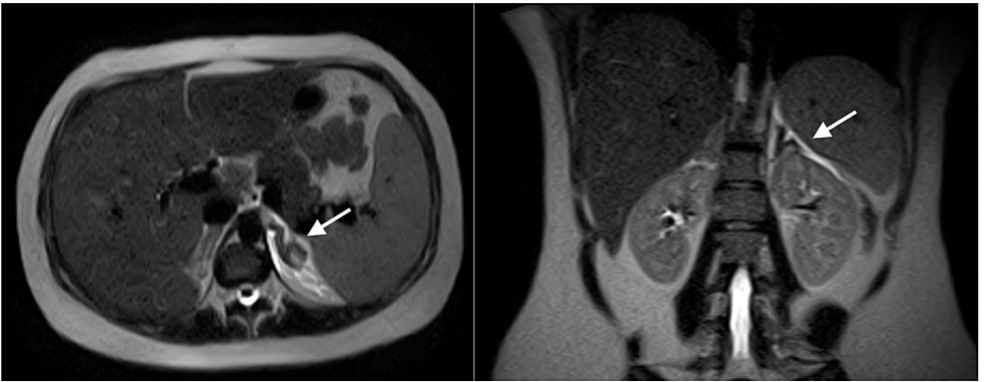
Unilateral left adrenal infarction with necrosis, and without hemorrhage

Despite IV fluids, antiemetics, and analgesia, the patient reported no change in pain intensity. Given the persistence and severity of her pain with increasing concern for an acute abdomen in pregnancy, further radiologic imaging was deemed vital. We did not do a CT scan to avoid fetal radiation exposure. MRI of the abdomen revealed unilateral left adrenal infarction with necrosis, and without evidence of hemorrhage (Figure 1). There was also evidence of edema surrounding the superior pole of the left kidney with subtle signal abnormality, which can be consistent with a recent history of pyelonephritis. We established supportive care with symptomatic management and morphine for pain control. The patient was evaluated for hypercoagulability and adrenal insufficiency to further evaluate the etiology and consequences of her non-hemorrhagic adrenal infarction. 

The thrombophilia screening returned negative results for antinuclear antibodies, lupus anticoagulant, anticardiolipin antibody, and antiglycoprotein antibody. Factor VIII activity was elevated >200% (55-200%) and protein S activity was decreased to 54% (63-140%). Factor II, factor V Leiden, and protein C activity were within normal limits. Cosyntropin stimulation testing was suggestive of primary adrenal insufficiency. The patient was started on stress dose steroids with IV hydrocortisone 50 mg every 12 hours. She was weaned to physiologic steroid dosing with PO hydrocortisone 20 mg every morning and 10 mg every afternoon. She was also prescribed low molecular weight heparin (LMWH) until six weeks postpartum. The patient had eventual resolution of abdominal pain with a morphine patient-controlled analgesia (PCA) pump and initiation of anticoagulation.

She was weaned off steroids one month after discharge and continued to self-administer enoxaparin. The patient had an uneventful planned induction of labor and delivery at 39 weeks of gestation. The etiology of her adrenal infarction was thought secondary to her elevated plasma factor VIII activity. Anticoagulation was continued for six months postpartum. 

## Discussion

The acute abdomen refers to an acute intra-abdominal presentation of intense pain, rigidity, and tenderness in a manner that may warrant immediate surgical intervention. This condition is especially problematic in pregnancy as complications can harm the mother along with the fetus. Therefore, timely evaluation and management are vital as the presentation may result in the form of life-threatening conditions, including obstetric complications, sepsis, infarction, or obstruction. The differential diagnosis for acute abdomen in pregnancy include ectopic pregnancy, placental abruption, ovarian torsion, ruptured ovarian cyst, HELLP syndrome, intra-abdominal pregnancy, and uterine rupture; non-gynecological etiologies remain on the differential, including appendicitis, pancreatitis, cholecystitis, intestinal obstruction, peptic ulcer disease, urolithiasis, and pyelonephritis [2].

Adrenal infarction is one of the rarest causes of AAP, which presents with abdominal, flank, back, or chest pain with hypotension or shock, fever, nausea, vomiting, and abdominal rigidity or rebound tenderness. As the adrenal glands produce mineral- and glucocorticoids, patients may also present with adrenal insufficiency. With the differential of acute abdomen in pregnancy being broad, and the limitedness of the physical examination, abdominal ultrasound is the preferred first imaging modality in pregnancy; however, it is frequently inconclusive. Diagnosis of adrenal infarction can also be made by CT, but we would avoid CT in pregnant patients because it involves fetal radiation exposure. Similarly, contrast-enhanced MRI can diagnose adrenal infarction, but MRI contrast is usually better avoided in pregnant patients because of potential fetal toxicity. Therefore, non-contrast MRI is considered a safe study in pregnancy. MRI features of adrenal infarction include diffuse enlargement of the adrenal gland, decreased signal intensity on T2-weighted images, and edema in the adjacent retroperitoneum [3]. The available literature on adrenal infarction primarily consists of case reports, a few small retrospective case series, and older articles that assess the pathology of infarcted adrenals from various causes. These reports all propose that adrenal vein thrombosis is the initial insult to the adrenal gland, especially in the setting of a hypercoagulable state such as antiphospholipid antibody syndrome (APS), followed by hemorrhagic infarction. However, hemorrhage might not always occur [5]. One case reported the cause of unilateral adrenal infarction in pregnancy to be a complication of APS [2]. Another case reported the cause of unilateral adrenal infarction in pregnancy to occur secondary to methylenetetrahydrofolate reductase (MTHFR) C677T gene mutation, which causes increased homocysteine levels that confer a hypercoagulable state [6].

The proposed hypothesis for the cause of adrenal vein thrombosis in pregnancy builds upon Virchow’s triad, which states that venous thrombosis occurs due to a combination of venous stasis, endothelial damage, and activation of coagulation factors. The hypothesis suggests that adrenal vein thrombosis may occur due to local venous stasis or turbulence from contraction of the adrenal vein against a gravid uterus, in which shearing forces cause vessel damage and release of catecholamines that make it an optimal site of thrombosis [7].

Pregnancy itself leads to physiologic changes that increase the risk of venous thromboembolism, making pregnant women particularly susceptible to adrenal infarction. Specific physiologic changes of pregnancy that induce a hypercoagulable state include higher blood volume, decreased production of anticoagulant factors, increased circulating levels of estrogen, and venous stasis due to compression by the gravid uterus. Other risk factors for adrenal vein thrombosis include heparin-induced thrombocytopenia, factor V Leiden deficiency, and elevated factor VIII levels. Our patient had elevated factor VIII activity, which in particular has been associated with an increased risk of venous thrombosis [8]. Elevated factor VIII levels have also been implicated in an increased risk of arterial thrombosis in coronary artery disease and stroke. Although our patient had factor VIII levels within the range of what is expected during her stage of pregnancy, values of factor VIII above 150 IU/dL still confer greater than a fivefold increased risk of venous thrombosis [9].

The regulation of factor VIII is dependent upon multiple factors, including levels of von Willebrand factor (vWF) and blood type. Patients with the non-O blood group have higher levels of vWF and factor VIII than patients with blood group O. Levels of factor VIII and vWF are also more elevated in females compared to males, patients with elevated body mass index, high triglycerides, and diabetes mellitus [10]. However, only 50% of patients with persistently high levels of factor VIII levels also have elevated wVF levels, indicating that there may be other factors in addition to vWF that lead to elevated factor VIII levels [11].

Adrenal infarctions are typically hemorrhagic and bilateral. Patients with bilateral adrenal infarction typically present with shock secondary to acute adrenal insufficiency. Those with unilateral adrenal infarction typically maintain normal adrenal function due to the contralateral adrenal gland being unaffected. However, our patient with only unilateral adrenal infarction developed primary adrenal insufficiency. It is possible that this patient already had some degree of depressed adrenal function, and that when compounded by an acute adrenal infarction, had insufficient time for the contralateral adrenal gland to compensate. The acute loss of function of her unilaterally functioning adrenal gland could have led to a substantial enough decline in production capacity to cause an abnormal response to cosyntropin stimulation [5].

Treatment for adrenal infarction includes therapeutic anticoagulation to prevent further progression of adrenal infarction of the contralateral adrenal gland. The risks versus benefits of anticoagulation in a pregnant patient need to be considered and the decision should be individualized in each patient. Specifically, there should be consideration of risk conversion to adrenal hemorrhage and the risk of bleeding that can occur with vaginal or cesarean deliveries. Anticoagulation for adrenal infarction is recommended in the absence of hemorrhage [5].

## Conclusions

Unilateral adrenal infarction is a rare event during pregnancy. Therefore, a high clinical suspicion must exist, and visceral organ infarction should always be included on the differential for the acute abdomen during pregnancy. This patient had multiple factors that placed her at increased risk of adrenal infarction. Her baseline hypercoagulable state due to pregnancy, elevated factor VIII levels, and recent hospitalization for pyelonephritis and urosepsis could have contributed to unilateral adrenal infarction. Once hemorrhage is excluded, the patient should be started on therapeutic anticoagulation to prevent progression of adrenal infarct or infarction of the contralateral adrenal gland. Therefore, prompt recognition and treatment are paramount and can lead to successful outcomes.
